# Effect of Aphidicolin, a Reversible Inhibitor of Eukaryotic Nuclear DNA Replication, on the Production of Genetically Modified Porcine Embryos by CRISPR/Cas9

**DOI:** 10.3390/ijms23042135

**Published:** 2022-02-15

**Authors:** Sergio Navarro-Serna, Celia Piñeiro-Silva, Chiara Luongo, John Parrington, Raquel Romar, Joaquín Gadea

**Affiliations:** 1 Department of Physiology, International Excellence Campus for Higher Education and Research “Campus Mare Nostrum”, University of Murcia, 30100 Murcia, Spain; sergio.navarro3@um.es (S.N.-S.); celia.pineiros@um.es (C.P.-S.); chiara.luongo@um.es (C.L.); rromar@um.es (R.R.); 2 Institute for Biomedical Research of Murcia (IMIB-Arrixaca), 30120 Murcia, Spain; 3 Department of Pharmacology, University of Oxford, Oxford OX1 3QT, UK; john.parrington@pharm.ox.ac.uk

**Keywords:** CRISPR/Cas9, DNA replication, mosaicism, pig, aphidicolin, gene editing, knock-out, TPCN1, embryo, in vitro fertilization

## Abstract

Mosaicism is the most important limitation for one-step gene editing in embryos by CRISPR/Cas9 because cuts and repairs sometimes take place after the first DNA replication of the zygote. To try to minimize the risk of mosaicism, in this study a reversible DNA replication inhibitor was used after the release of CRISPR/Cas9 in the cell. There is no previous information on the use of aphidicolin in porcine embryos, so the reversible inhibition of DNA replication and the effect on embryo development of different concentrations of this drug was first evaluated. The effect of incubation with aphidicolin was tested with CRISPR/Cas9 at different concentrations and different delivery methodologies. As a result, the reversible inhibition of DNA replication was observed, and it was concentration dependent. An optimal concentration of 0.5 μM was established and used for subsequent experiments. Following the use of this drug with CRISPR/Cas9, a halving of mosaicism was observed together with a detrimental effect on embryo development. In conclusion, the use of reversible inhibition of DNA replication offers a way to reduce mosaicism. Nevertheless, due to the reduction in embryo development, it would be necessary to reach a balance for its use to be feasible.

## 1. Introduction

The production of genetically edited animals became a reality in the 1980s when the first transgenic mice were obtained by recombinant plasmid pronuclear microinjection [1]. In 1985, the generation of the first genetically edited pigs by random insertion of foreign DNA was reported [2,3].

Subsequently, the development and application of programmable endonucleases—such as zinc finger nucleases (ZFNs) [4], transcription activator-like effector nucleases (TALENs) [5], and clustered regularly interspaced short palindromic repeat (CRISPR)/CRISPR-associated protein (Cas) [6,7,8]—led to a revolution in the production of genetically modified animals. The mechanism of these endonucleases consists in the generation of double-strand breaks in target DNA which can be repaired by two different mechanisms: non-homologous end joining (NHEJ), which allows the generation of insertions-deletions (INDELs) in the DNA sequence that can produce a knock-out (KO) allele, or homology-directed repair (HDR), which can allow the replacement of the wildtype (WT) sequence with a desired sequence that is introduced via a foreign DNA fragment, generating a KO or knock-in (KI) allele [9].

The use of programmable endonucleases removed a major obstacle for genetic modification of non-rodent mammalian species, which had only been possible through the random insertion of foreign DNA [10], or use of homologous recombination of cultured cells and subsequent somatic cell nuclear transfer (SCNT). The application of these nucleases also represented a substantial increase in the mutation efficiency in mice and other mammals, making the rapid and efficient generation of genetically modified animals possible [10].

Currently, the CRISPR/Cas9 system is the most widely used gene editing tool. Its first application in the porcine species was reported in 2014, when CRISPR/Cas9 was applied to generate pigs both by SCNT [11] and by direct embryo editing [12].

From the first use of the CRISPR/Cas9 system until the present, genetically modified pigs have been generated for different applications including furthering understanding of the role of genes in basic science studies [13,14,15]; the editing of genes for application in animal health programs such as increasing resistance to diseases like porcine reproductive and respiratory syndrome (PRRS) [16,17]; the improvement of animal characteristics for animal production such as improving muscle development [18,19], or modifying the fatty acid profile [20]; and the use of pigs for biomedical purposes such as creating models of human disease [21,22,23], or the generation of organs for xenotransplantation [24,25].

Although the use of programmable endonucleases like CRISPR/Cas9 has led to a great improvement in the generation of genetically edited animals, there are still limitations such as off-target editing and mosaicism. The first does not seem to be a serious problem because RNA-guide design programs allow the selection of those guides that have a low chance of off-target mutations and therefore only a few number of cases have been reported in the bibliography (reviewed by Navarro-Serna et al., 2020) [10].

Instead, mosaicism is the most important limitation for gene editing in embryos [12]. It involves the presence of more than one cell type in the same individual with more than two different alleles of the same gene [14]. This problem takes place when the activity of programmable endonucleases such as the CRISPR/Cas9 system generates insertions or deletions (INDELs) after the first DNA replication in zygotes or even after subsequent cell divisions. The generation of mosaic organisms reduces the possibility of producing a KO organism in the first generation because not all INDELs produce KO alleles [14]. Moreover, mosaicism creates the possibility that edited alleles are only present in somatic cells and not in the germ line, and therefore animals with such characteristics are no use for the transmission of the desired allelic changes to offspring. Mosaicism is thus associated with a longer time for generating the designed models and a significant increase in cost of the projects.

**Figure 1:**
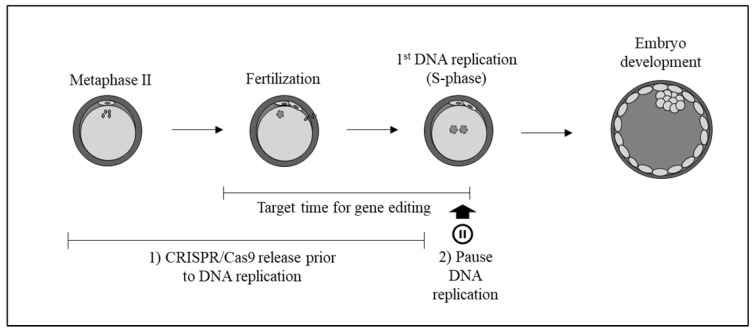
Possible time-related strategies to decrease mosaicism: (1) Injection of CRISPR/Cas9 to act before DNA replication. (2) Use of inhibitors to temporarily pause DNA replication thus increasing the time for the CRISPR/Cas9 system to work.

A possible strategy to reduce mosaicism would be to try and ensure that the CRISPR/Cas9 system edits the DNA before the zygote enters S phase, when DNA replication occurs (Figure 1). In previous research from our group, we observed that the delivery of the CRISPR/Cas9 system by microinjection before the first DNA replication in zygotes reduced the mosaicism rate [14]. Therefore, another possible strategy would be to slow down or pause DNA replication to increase the time over which the CRISPR/Cas9 system acts without leading to the presence of more than two alleles.

Aphidicolin is a reversible inhibitor of eukaryotic nuclear DNA replication and blocks the cell cycle at the pre-S phase [26]. The reversible nature of this drug allows those cells subjected to it to remain viable. The use of aphidicolin has been reported in cell culture, including in cultured cells from pigs, to synchronize somatic cells for the generation of embryos by SCNT [26,27,28,29]. Its use was also reported on embryos of different species such as echinoderma [30], murine [31,32,33,34], and bovine [35,36] embryos, in which a satisfactory inhibition of DNA replication was achieved. As far as we are aware, there is no published reference to the use of aphidicolin directly in porcine oocytes/embryos for inhibition of DNA replication. Additionally, there is no literature about its employment for reducing mosaicism after CRISPR/Cas9 treatment. Both aspects are explored in this study for the first time.

We hypothesized that a delay of DNA replication by the addition of aphidicolin in the generation of genetically modified porcine embryos could reduce the mosaicism rate without reducing embryo production, thus obtaining a final improvement of the system.

To achieve this goal, because the use of aphidicolin in porcine embryos has not been previously reported, the first step was to determine the concentration that allows the reversible inhibition of DNA replication and then to evaluate possible detrimental effects of these concentrations on embryo development.

Subsequently, the objective was to evaluate if the application of aphidicolin makes it possible to improve the gene editing system by reduction of mosaicism without affecting the quality and quantity of genetically modified embryos obtained. For this, we used single guide RNAs (sgRNAs) against TPCN1 and embryos were subjected to different concentrations of CRISPR/Cas9 and different methods to deliver CRISPR/Cas9.

## 2. Results

**Figure 2:**
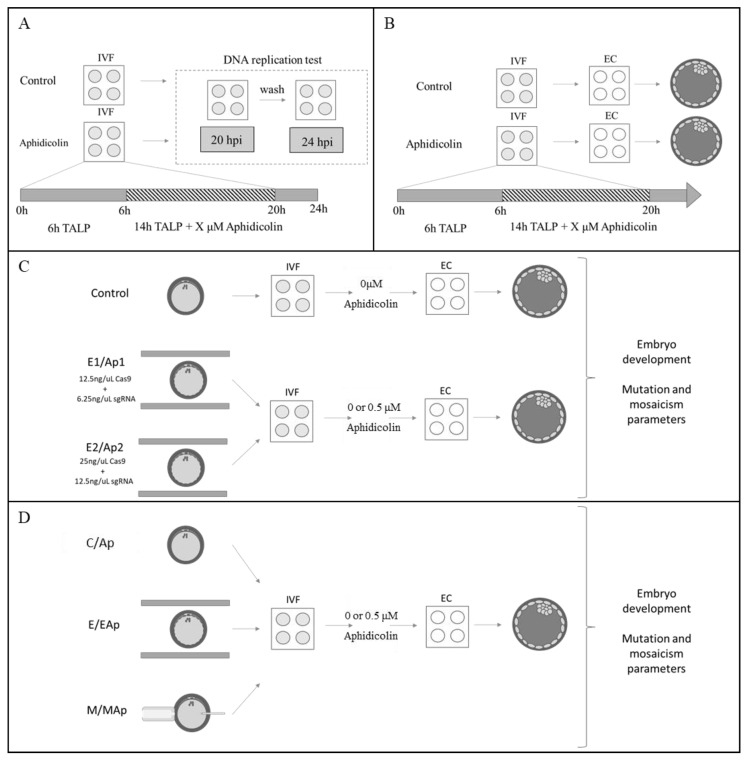
Experimental design. (**A**) Effect of aphidicolin on reversible inhibition of DNA replication. In vitro-derived zygotes (IVF) were cultured with different concentrations of aphidicolin or without aphidicolin from 6 to 20 h after insemination (hpi). Half of them were fixed 20 h after insemination and the other half were cultured in NCSU23a medium and fixed 4 h later. DNA replication was evaluated in all groups. (**B**) Effect of aphidicolin on porcine embryo development. IVF zygotes were cultured during the same time conditions with different concentrations of aphidicolin and then subjected to embryo culture (EC) for 6.5 days until blastocyst stage. (**C**) Effect of aphidicolin and ribonucleoprotein (RNP) concentration on gene editing. Oocytes were electroporated with two concentrations of RNP against TPCN1 and zygotes were in vitro cultured for 6.5 days until blastocyst stage in the presence or absence of aphidicolin. Embryo development and mutation parameters were evaluated. (**D**) Effect of aphidicolin and methodology in gene editing. Oocytes were electroporated or microinjected with RNP against TPCN1 and zygotes were in vitro cultured in the presence or absence of aphidicolin. Embryo development and mutation parameters were evaluated at 6.5 days after culture. Six groups were used for this experiment: C (control), Ap (0.5 μM of aphidicolin treatment), E (electroporated), EAp (electroporated with 0.5 μM of aphidicolin treatment), M (microinjected), and MAp (microinjected with 0.5 μM of aphidicolin treatment).

The experimental design is depicted in Figure 2 to help in reading of the manuscript.

### 2.1. Effect of Aphidicolin on Reversible Inhibition of DNA Replication in Porcine Zygotes

**Figure 3:**
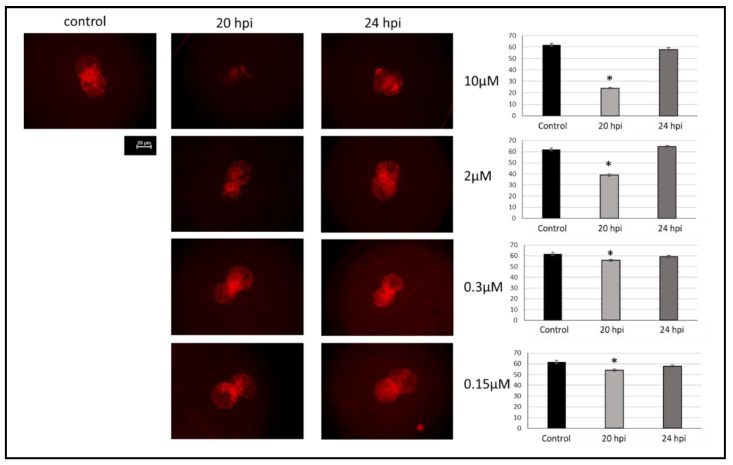
Evaluation of the effect of aphidicolin on reversible inhibition of DNA replication in porcine zygotes. In vitro derived zygotes were fixed and stained 20 h after insemination (20 hpi) after being cultured without aphidicolin (Control) and with different concentrations (0.15, 0.3, 2, and 10 µM) of aphidicolin. Half of them were washed and in vitro cultured without aphidicolin for 4 h, and then fixed (24 hpi). Images of zygote pronuclei stained with Click-iT™ EdU Alexa Fluor™ Imaging Kit assessed at 20 and 24 hpi are shown. Red fluorescence shows DNA after replication. Images were processed with Image J software (NIH) and relative fluorescence was assessed. Fluorescence intensity was relativized with respect to the pronucleus with the highest signal intensity. Comparison between the control group and zygotes 20 hpi and 4 h after removing aphidicolin (24 hpi) was performed. * Indicates significant differences with the other groups at a specific aphidicolin concentration (*p* < 0.05). Scale bar: 20 μm.

As shown in Figure 2A and Figure 3, incubation of zygotes with aphidicolin at different concentrations reduces the degree of DNA replicated at 20 hpi with significant differences (*p* < 0.05) with respect to control embryos. The reduction in DNA replication increased with the concentration of aphidicolin. Furthermore, all zygotes in all groups recovered their DNA replication level after 4 h without aphidicolin, reaching fluorescence levels similar to the control group thus confirming the reversibility of the inhibitor.

### 2.2. Effect of Aphidicolin on Porcine Embryo Development

**Figure 4:**
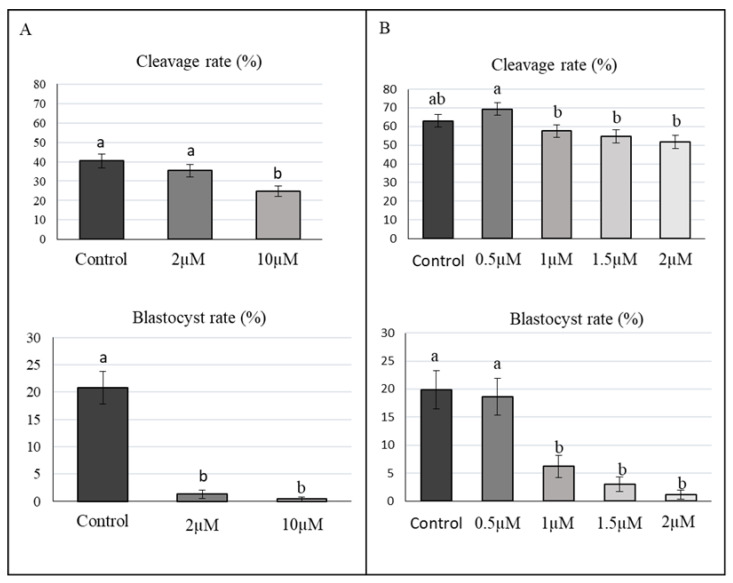
Effect of aphidicolin in porcine embryo development. (**A**) Cleavage and blastocyst rates for in vitro derived porcine embryos cultured with 0 (control), 2 and 10 µM aphidicolin. (**B**) Cleavage and blastocyst rate of in vitro derived porcine embryos cultured with 0 (control), 0.5, 1, 1.5, and 2 µM aphidicolin. Cleavage rate (%): Two-cell embryos per total number of inseminated oocytes. a,b Values with different superscripts are significantly different (*p* < 0.05).

In an initial experiment, putative zygotes were incubated in the presence of 2 or 10 µM aphidicolin (Figure 2B and Figure 4A), whereas the cleavage rate was decreased only at 10 µM aphidicolin; the blastocyst rate markedly decreased at both concentrations, showing that aphidicolin at these levels had a significant toxic effect on further embryo development. Subsequently, lower concentrations of aphidicolin (ranging from 0.5 to 2 µM) were tested. In this subsequent experiment (Figure 4B), the cleavage rate was over 50% in all groups with non-significant effects of incubation with aphidicolin. As observed in the first experiment, the blastocyst rate was affected, being significantly lower in groups incubated with 1, 1.5, and 2 µM of aphidicolin with respect to the control group. However, the use of 0.5 µM aphidicolin did not produce a toxic effect, with embryos reaching the same blastocyst rate as the control group (around 20%), so this concentration was used for the subsequent experiments.

### 2.3. Effect of Aphidicolin and RNP Concentration on Gene Editing

**Figure 5:**
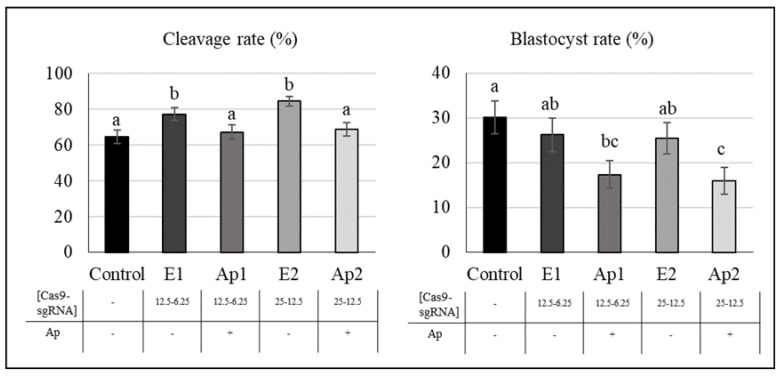
Effect of aphidicolin and ribonucleoprotein concentration in porcine embryo development. Figure shows cleavage and blastocyst rate of in vitro matured oocytes electroporated with 12.5 ng/µL Cas9 protein plus 6.25 ng/µL sgRNA and incubated without (E1) or with 0.5 μM aphidicolin (Ap1), electroporated with 25 ng/µL of Cas9 protein and 6.25 ng/µL of sgRNA and incubated without (E2) or with aphidicolin (Ap2), and Control group without electroporation or aphidicolin. Ap: aphidicolin; Cleavage rate (%): Two-cell embryos per total number of inseminated oocytes. a–c Values with different superscripts are significantly different (*p* < 0.05).

As shown in Figure 2C and Figure 5, the cleavage rate was higher in electroporated groups without aphidicolin treatment with respect to the other groups (*p* < 0.01) (Figure 5). The blastocyst rate was similar in electroporated and control embryos, but this rate significantly decreased in groups treated with aphidicolin and electroporated (Ap1 and Ap2) with respect to the control group (*p* = 0.01). This difference was higher in the oocytes electroporated with the highest concentration of CRISPR/Cas9 RNP (Ap2), which showed significative differences with respect to the other groups not treated with aphidicolin. In two-way ANOVA, significant differences were found in the cleavage and blastocyst rates that were only related to the incubation with aphidicolin (*p* < 0.01, Table 1), being lower in aphidicolin treatment groups, while differences in RNP concentration and interaction of aphidicolin and RNP concentration did not lead to significant differences in cleavage and blastocyst rates (Table 1).

In terms of mutation parameters (Table 2), the mutation rate was higher in groups with a higher RNP concentration, and no differences were found following the use of aphidicolin in comparison with their controls. Significant differences were found in mutation rates (*p* = 0.01), but these were only related to RNP concentration and not related to aphidicolin treatment, as was also seen in the two-way ANOVA analysis (Table 1). In addition, a tendency (*p* = 0.06) was found in the percentage of mosaic embryos with respect to the total number of embryos (Table 2). This tendency was also observed in the two-way ANOVA analysis (Table 1), where a lower percentage of mosaic embryos was observed in the groups treated with aphidicolin. Other parameters did not show significant differences.

### 2.4. Effect of Aphidicolin and Methodology on Gene Editing (Electroporation vs. Microinjection)

**Figure 6:**
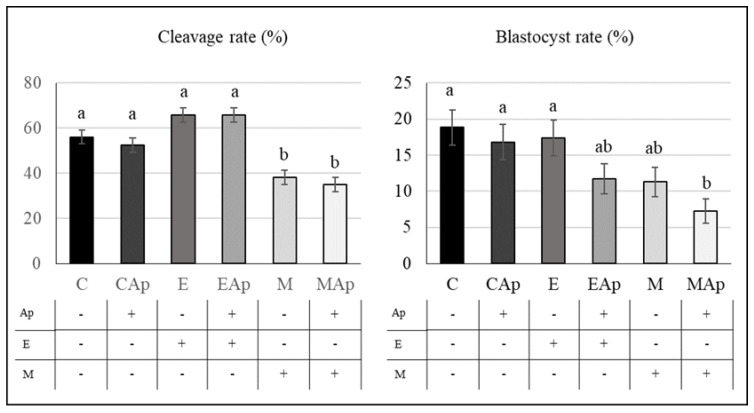
Effect of aphidicolin and method of CRISPR/Cas9 delivery on porcine embryo development. Figure shows cleavage and blastocyst rate of oocytes incubated without (C) or with 0.5 μM aphidicolin (Ap), electroporated and incubated without (E) or with 0.5 μM aphidicolin (EAp) and microinjected and incubated without (M) or with 0.5 μM aphidicolin (MAp). a,b Values with different superscripts are significantly different (*p* < 0.05).

During embryo development (Figure 2D and Figure 6), the cleavage rate was not affected by the treatment with aphidicolin, however it was affected by the methodology used to deliver CRISPR/Cas9 (Table 3), being significantly lower in groups that were microinjected with respect to other groups (*p* < 0.01, Figure 6, Table 3). The blastocyst rate was similar in all groups except in groups microinjected with aphidicolin (MAp), where the blastocyst rate was lower (*p* < 0.01) than C, Ap, and E groups (Figure 6). In two-way ANOVA (Table 3), blastocyst rate was affected by both aphidicolin incubation (*p* = 0.03) and methodology (*p* < 0.01), being lower in groups treated with aphidicolin and in groups that were microinjected.

Regarding mutation parameters for TPCN1 (Table 4), no significant differences were found between groups for any parameter. In two-way ANOVA (Table 3), the percentages of mosaicism with respect to the total number of embryos, and mosaicism with respect to the number of mutant embryos, were affected by the incubation with aphidicolin (*p* = 0.03 and *p* = 0.05, Table 4), being lower in groups incubated with aphidicolin. That fact confirms the efficiency for reducing mosaicism rate, although the final efficiency of the system, measured as the rate of biallelic KO embryos derived from oocytes, was not different (Table 3 and Table 4).

## 3. Discussion

Mosaicism is the main problem related to the production of genetically edited animals when gene editing is performed in embryos and not in somatic cells before performing SCNT [37]. Different strategies have been carried out to try to reduce mosaicism, all of them based on time factors, to try to generate INDELs before the first DNA replication [14,38,39]. Among these strategies, the use of modified Cas9 protein with ubiquitin-proteasomal degradation signals to reduce the half-life of the RNP, has been employed [39]. The use of three-prime repair exonuclease 2 (mTrex2) in porcine zygotes to shorten the time of DNA repair after cuts generated by the CRISPR/Cas9 system with the objective of repairing the DNA chain before replication has also been reported [38]. The last reported strategy consisted of releasing CRISPR/Cas9 into the embryo as early as possible, even microinjecting oocytes before they were inseminated [14]. Mosaicism rate was reported as being reduced in all these strategies, thus all results confirmed that the timing of editing with respect to the first DNA replication is a key factor that affects mosaicism.

Until now, the use in zygotes of reversible DNA inhibitors, such as aphidicolin, to try to reduce the degree of mosaicism, has not been described. Although the use of aphidicolin had already been described in porcine cells, it was necessary to verify its effectiveness in embryos [26,27,28,29], and also in embryos of different species for studies of DNA replication [30,31,32,33,34,35]. In previous research, we observed that the first DNA replication in in vitro produced embryos starts between 8–9 h post-insemination [14]. Therefore, we decided to add aphidicolin 2–3 h before the beginning of DNA replication to be able to stop the cell cycle before S-phase. Our results showed that the use of aphidicolin to treat zygotes for up to 20 h post insemination resulted in lower DNA replication values than in normal conditions. Previous studies showed that the toxicity of aphidicolin depends on concentration and time of incubation [32,33]. In our study, we needed the embryos to be exposed to inhibition for a limited time, so we focused on modifying the concentration. Unfortunately, most effective concentrations tested were not compatible with embryo development. Despite this, we managed to work with low concentrations of aphidicolin that were compatible with embryo viability.

The minimal concentration of aphidicolin required to produce reversible inhibition of DNA replication without toxic effects was found in this study to be 0.5 µM. Reversibility of the inhibition has been shown, and the inhibition of DNA synthesis occurred efficiently and persisted with all the concentrations of aphidicolin up to 10 µM, until removal of the inhibitor. The toxic effect of aphidicolin on cleavage rate in our study was lower, in contrast to previous results observed in bovine zygotes [36]. The toxic effect on embryo development could be related to the decrease in transcriptional and translational activity during the first rounds of DNA replication that are important regulators of early gene expression [35]. Although incubation with aphidicolin has been shown to halve the rate of mosaicism, the toxicity of this compound also reduced the blastocyst development rate. Therefore, overall, the rate of non-mosaic KO embryos produced with or without aphidicolin was similar. Therefore, under the tested conditions, the use of aphidicolin was not advantageous. However, we think the use of this strategy (use of aphidicolin) could be useful for other models/labs under other conditions, where the percentage of mosaicism is different or once the embryo development is improved for porcine blastocysts, or using aphidicolin in a different manner (with different concentration and time of coculture with the oocytes), because the toxicity of aphidicolin is concentration and time dependent [32,33].

The concentration of CRISPR/Cas9 is another factor related to the mutation and mosaicism rates. A high concentration of RNP improves the percentage of blastocysts with biallelic mutations, thus decreasing the percentage of heterozygous or mosaic embryos [40,41]; however, a high RNP concentration can be toxic for embryo development [41]. Due to this, the concentration of CRISPR/Cas9 must be optimized for each sgRNA and even for each species [37]. In this study, we have observed that increasing the CRISPR/Cas9 concentration also increased the mutation rate, however, a decrease in mosaicism was not observed.

Even though previous studies reported that different methods of CRISPR/Cas9 delivery such as intracytoplasmic microinjection and electroporation do not produce critical damage in terms of embryo development [14,42], it was necessary to check this, and also whether the concomitant use of aphidicolin could generate toxic effects. Although the decrease in the blastocyst rate was not significant, a toxic effect of the use of aphidicolin was observed after electroporation or microinjection. This effect was not observed in control embryos treated with aphidicolin, perhaps because the manipulation required for gene editing makes embryos more susceptible to toxicity from compounds in the environment.

In this study, an effect of the method of CRISPR/Cas9 delivery on embryo development was observed, and this may be because the concentration of RNP delivered by microinjection is higher than by electroporation and a toxic concentration could be reached [41]. However, the mutation rate was similar in terms of the percentage of mutant embryos that reached blastocyst stage.

## 4. Materials and Methods

### 4.1. Ethical Issues

The study was developed according to the Spanish Policy for Animal Protection (RD 53/2013), which conforms to the European Union Directive 2010/63/EU regarding the protection of animals used in scientific experiments. This project was positively evaluated by the Ethics Committee at the University of Murcia and Murcia Regional Government for the use of Genetically Modified Organisms (Reference A/ES/16/79).

### 4.2. Culture Media Reagents

All chemicals were purchased from Sigma-Aldrich Quimica, S.A. (Madrid, Spain) unless otherwise indicated.

### 4.3. Design of Single Guide RNAs

**Figure 7:**
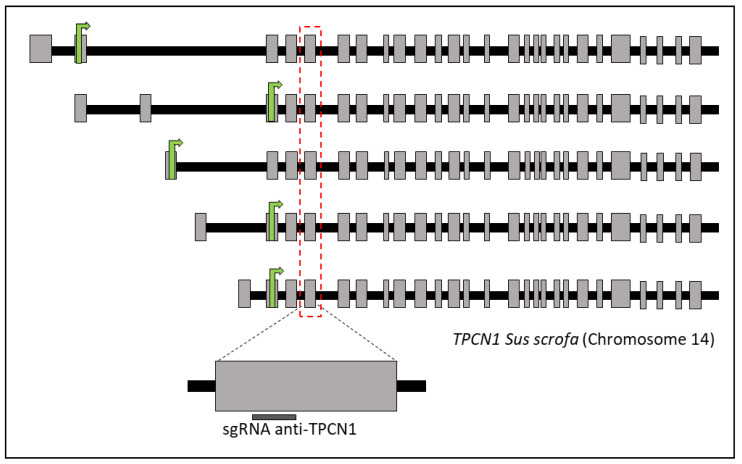
Schematic of TPCN1 isoforms described in Sus scrofa (NC_010456.5). Gray boxes represent exons and black boxes introns. The green arrow shows the exon containing the start codon. The red dotted line shows exon 9, where the target region is located.

Single guide RNAs (sgRNAs) against TPCN1 (CATTCGGCACAAACGGACCA) were designed using Braking-Cas software [43] (BioinfoGP, CNB-CSIC, Madrid, Spain). As shown in Figure 7, exon 9 is selected to edit all TPC1 isoforms described in the porcine genome. Both sgRNA and Cas9 protein were purchased from IDT (Integrated DNA Technologies, Leuven, Belgium).

### 4.4. In Vitro Maturation (IVM)

Cumulus–oocyte complexes (COCs) were obtained from gilt ovaries from the slaughterhouse and processed as previously described [44]. Briefly, ovaries were transported in saline solution at 38 °C and once in the lab these were washed once in 0.04% cetrimide solution and twice in saline solution at 38 °C. COCs were collected from aspiration of follicles between 3–6 mm diameter, selected under a stereomicroscope, and washed in Dulbecco’s PBS (DPBS) with 1 mg/mL polyvinyl alcohol (PVA) and then in maturation medium (NCSU37) [45]. After washing, groups of 50–55 COCs were cultured in 500 µL NCSU37 supplemented with 10% (*v*/*v*) porcine follicular fluid, 1 mM dibutyryl cAMP, 10 UI/mL eCG and 10 UI/mL hCG, and cultured for 20–22 h at 38.5 °C and 5% CO_2_ followed by an additional 20–22 h in NCSU37 without dibutyryl cAMP, eCG, and hCG.

### 4.5. CRISPR/Cas9 Electroporation

After IVM, 50 µL hyaluronidase at 0.5% was added to each well of NCSU37 and COCs were incubated for 5 min, then matured COCs were mechanically decumulated with a micropipette until most of the cumulus cells were removed [14].

Before electroporation, oocytes were washed in Opti-MEM I Reduced Serum Media (Thermofisher, Waltham, MA, USA). Subsequently, groups of 100 oocytes were transferred to a drop containing CRISPR/Cas9 ribonucleoprotein (RNP) and were put in a slide between 1 mm gap electrodes (45-0104, BTX, Harvard Apparatus, Holliston, MA, USA) connected to an ECM 830 Electroporation System (BTX, Harvard Apparatus, Holliston, MA, USA). Finally oocytes were electroporated using 4 pulses of 30 V, at 1 msec pulse duration, and 100 ms pulse interval [46].

### 4.6. In Vitro Fertilization (IVF)

Procedures for IVF were mainly the same as described previously [44]. In vitro matured oocytes were washed in TALP medium [47] supplemented with 1 mM sodium pyruvate, 0.3% BSA, and 50 µg/mL gentamycin (IVF-TALP), and transferred in groups of 50–55 oocytes to each well containing 250 µL IVF-TALP medium. Oocytes were inseminated with frozen–thawed ejaculated spermatozoa from a fertile boar that had been selected by a swim-up procedure [48]. One 0.25 mL-straw was thawed in a water bath (30 s, 38 °C) and semen diluted in 2 mL NaturARTsPIG sperm swim-up media (Embryocloud, Murcia, Spain) at 38 °C. Sperm selection was performed by adding 1 mL sperm swim-up media in a conical tube and 1 mL thawed-diluted sperm to the bottom of the tube. Tubes were then incubated (38 °C, 20 min, 45° angle), 500 µL of the top medium were aspirated, the sperm concentration was adjusted to 3000 cells/mL, spermatozoa were diluted in IVF-TALP, and oocytes inseminated with 250 µL sperm solution (final IVF well volume 500 µL). Gametes were cocultured at 38.5 °C, 5% CO_2_, and 7% O_2_, for 20–22 h.

### 4.7. In Vitro Embryo Culture (EC)

After gamete co-incubation in IVF-TALP for 18–20 h, putative zygotes were cultured in NCSU23a (NCSU23 medium supplemented with 5 mM sodium lactate, 0.5 mM sodium pyruvate, and essential (1% *v*/*v*) and nonessential (1% *v*/*v*) aminoacids) [44] and cultured for 24 h at 38.5 °C, 5% CO_2_, and 7% O_2_. After this, cleavage rate was evaluated, and 2–4 cell embryos were transferred to NCSU23b (NCSU23 medium supplemented with 5.5 mM glucose and essential (1% *v*/*v*) and nonessential (1% *v*/*v*) aminoacids) [44] until 6.5 days after insemination. After in vitro culture, blastocyst development rate was evaluated (blastocysts/oocytes) and blastocysts were collected to evaluate mutation as described in Section 4.9.

### 4.8. DNA Replication Test

DNA replication was analyzed using a Click-iT™ EdU Alexa Fluor™ Imaging Kit (Invitrogen, Waltham, MA, USA) as previously described [14]. At the precise insemination time, 2.5 µL of 10 mM stock solution of EdU was added to each IVF-TALP well and all groups of in vitro derived zygotes were fixed in 4% paraformaldehyde in DPBS-PVA for 30 min at room temperature. Samples were then permeabilized (0.1% Triton-X in PBS for 15 min), washed three times in PBS, incubated for 15 min in Click-iT™ reaction cocktail prepared according to the manufacturer’s instructions, washed three times, and stained with Hoechst 33342 (10 µg/mL, 30 min). Finally, samples were mounted on slides using mounting medium (DPBS-PVA, glycerol and 10 µg/mL Hoechst 1:1:1 *v*/*v*) and DNA replication was evaluated by epifluorescence microscopy. Red fluorescence showed DNA after replication. Images were processed with Image J software (NIH) and relative fluorescence was assessed [49]. Fluorescence intensity was relativized with respect to the pronucleus with the highest signal intensity.

### 4.9. Mutation Analysis

Zonae pellucidae of blastocysts were digested with 0.5% pronase (Protease from Streptomyces griseus, Sigma-Aldrich, Madrid, Spain), and ZP-free blastocysts washed in nuclease free water and stored individually with minimum volume at −80 °C until analysis. DNA extraction and PCR were performed using a Phire Animal Tissue Direct PCR Kit (Thermofisher, Waltham, MA, USA). Genomic DNA was extracted following the dilution protocol of this kit. One microliter sample was used for a 12.5 µL PCR reaction mix containing 0.5 µM primers. The PCR cycling times were 5 min at 98 °C, followed by 35 cycles (denaturation 5 s at 98 °C, annealing 5 s at 64.7 °C) and a final extension for 1 min at 72 °C.

Mutation detection was performed by a fluorescent PCR-capillary gel electrophoresis technique [14,50]. PCR was carried out using 6-FAM-labeled forward primers. After PCR, samples were diluted 1:100 *v*/*v* in TE buffer and 1 µL of the mixed samples was added to a clean Eppendorf containing 11.5 µL Hi-DiTM formamide (Thermofisher, Waltham, MA, USA) and 0.1 µL GeneScanTM 500 LIZ Size Standard (Applied Biosystem, Thermofisher, Waltham, MA, USA). The sample was incubated (3 min at 95 °C), immediately chilled on ice for 2 min, and analyzed by capillary gel electrophoresis on a 3500 Genetic Analyzer (Applied Biosystems, Thermofisher, Waltham, MA, USA). The details of the instrumental protocol were similar to that previously described [50]: capillary length: 50 cm; polymer: POP7; dye set: G5; run voltage: 19.5 kV; pre-run voltage: 15 kV; injection voltage: 1.6 kV; run time: 1330 s; pre-run time: 180 s; injection time: 15 s; data delay: 1 s; size standard: GS500 (−250) LIZ; size-caller: SizeCaller v1.10. Results were analyzed using Gene Mapper 5 (Life Technologies, Carlsbad, CA, USA).

Samples were considered to be wild type when the peak obtained by capillary electrophoresis was the same size as the control peak. Other peaks of different sizes with respect to the control peak were considered to be mutant, and when more than two peaks were detected in a sample it was considered as mosaic.

### 4.10. Statistical Analysis

Data are expressed as mean ± SEM. The variables in all experiments were tested for their normality by a Shapiro–Wilk test. Data that were not normally distributed were analyzed by a Kruskal–Wallis test. When data showed significant differences (*p* < 0.05), values were compared by a Conover–Inman test for pairwise comparisons. Parameters without normal distribution were cleavage rate, blastocyst rate, mutation rate, mosaicism/total, number of alleles, mosaicism/mutant, and biallelic KO/total. Data with a normal distribution were analyzed by one-way analysis of variance (ANOVA). A parameter with normal distribution was relative fluorescence of DNA replication. When data showed significant differences (*p* < 0.05), values were compared by a pairwise multiple comparison post hoc test (Tukey). Two-way ANOVA was also performed to evaluate the synergistic effect of aphidicolin treatment with the RNP concentration and the methodology. All data analysis was performed using SYSTAT 13.

### 4.11. Experimental Design

**Experiment 1.** Effect of aphidicolin on reversible inhibition of zygote DNA replication.

To evaluate whether the treatment with aphidicolin can produce reversible inhibition of DNA replication in zygotes, aphidicolin was added at different concentrations (control (0 µM), 0.15, 0.30, 2, and 10 µM) to the IVF medium 6 h post insemination (hpi) (Figure 2A) and putative zygotes were cultured in this medium until 20 hpi. Subsequently, half of them were fixed to evaluate DNA replication and the other half were washed to eliminate the aphidicolin and cultivated in NCSU23a without aphidicolin for an additional 4 h and then fixed to observe if DNA replication was restored. DNA replication was evaluated at 20 and 24 hpi. Three replicates with a total of 40–60 zygotes per group were analyzed.

**Experiment 2.** Effect of aphidicolin on porcine embryo development.

To evaluate the effect on embryo development (Figure 2B), aphidicolin was added to the IVF medium 6 hpi at two different concentrations, 2 and 10 µM, and compared with a control group (0 µM). Putative zygotes were subsequently cultured at 20 hpi and cleavage and blastocyst development rates were recorded during the following days. Due to the toxic effects on embryo development, a second experiment was performed with lower concentrations of aphidicolin: control (0 µM), 0.5, 1, 1.5, and 2 µM of aphidicolin. Four replicates with 50–55 oocytes per replicate and group were performed.

**Experiment 3.** Effect of aphidicolin and RNP concentration in gene editing by electroporation.

To analyze the effect of ribonucleoprotein (RNP) concentration and the use of aphidicolin in the generation of KO embryos (Figure 2C), oocytes were electroporated using a CRISPR/Cas9 complex targeted against TPCN1. Oocytes were electroporated at two different concentrations of RNP: 12.5 ng/µL Cas9 protein plus 6.25 ng/µL sgRNA or 25 ng/µL Cas9 protein plus 6.25 ng/µL sgRNA. At 6 hpi putative zygotes were cultured with 0 or 0.5 µM aphidicolin until 20 hpi. A total of five groups were evaluated: C (control without aphidicolin nor electroporation), E1 (electroporated, without aphidicolin and the lowest concentration of RNP), Ap1 (electroporated, with aphidicolin and the lowest concentration of RNP), E2 (electroporated, without aphidicolin and the highest concentration of RNP), and Ap2 (electroporated, with aphidicolin and the highest concentration of RNP). Three replicates with 50–55 oocytes per replicate and group were performed.

**Experiment 4.** Effect of aphidicolin and methodology in gene editing.

To analyze the effect of aphidicolin incubation combined with different methods to deliver CRISPR/Cas9 system into the oocyte (Figure 2D), an sgRNA against TPCN1 was also used. In this experiment oocytes were microinjected or electroporated with or without aphidicolin. Subsequently, at 6 hpi, putative zygotes were cultured with 0 or 0.5 µM aphidicolin until 20 hpi. A total of six groups were evaluated: C (control without aphidicolin nor RNP), Ap (with aphidicolin and without RNP), E (electroporated without aphidicolin and with RNP), EAp (electroporated with aphidicolin and RNP), M (microinjected without aphidicolin), and Map (microinjected with aphidicolin). Four replicates with 50–55 oocytes per replicate and group were performed.

## 5. Conclusions

The possibility of delaying the DNA replication time provides another possible way to try to reduce mosaicism in gene edited embryos. Although the incubation with aphidicolin leads to a decrease of mosaicism, it can also compromise embryo development, so it would be necessary to reach a balance for its use to be feasible.

## Figures and Tables

**Table 1:** Two-way ANOVA analysis for the effect of aphidicolin and ribonucleoprotein (Cas9/sgRNA) concentration.

	Aphidicolin	[Cas9/sgRNA]	Aphidicolin × [Cas9/sgRNA]
Cleavage rate ^1^	<0.01	0.23	0.41
Blastocyst rate ^2^	<0.01	0.75	0.93
Mutation rate ^3^	0.09	<0.01	0.52
Mosaicism/total ^4^	0.06	0.15	0.39
Number of alleles ^5^	0.13	0.89	098
Mosaicism/mutant ^6^	0.18	0.68	0.78
Biallelic KO/total ^7^	0.81	0.53	0.91

^1^ Two-cell embryos per total number of inseminated oocytes. ^2^ Blastocysts obtained per total number of inseminated oocytes. ^3^ Percentage of embryos with some mutant alleles. ^4^ Percentage of mutant embryos with more than 2 alleles with respect to total embryos. ^5^ Mean number of alleles per embryo. ^6^ Percentage of mutant embryos with more than 2 alleles with respect to mutant embryos. ^7^ Percentage of mutant embryos with both alleles mutated with respect to total embryos.

**Table 2:** Effect of ribonucleoprotein (Cas9/sgRNA) concentration and aphidicolin on mutation parameters expressed as mean ± SEM.

	E1	EAp1	E2	EAp2	*p* Value
[Cas9/sgRNA] (ng/µL)	12.5/6.75	12.5/6.75	25/12.5	25/12.5	
0.5 μM aphidicolin	-	+	-	+	
Mutation rate ^1^	48.78 ^a^ (20/41)	40.63 ^a^ (13/32)	76.47 ^b^ (39/51)	58.06 ^ab^ (18/31)	0.01
Mosaicism/total ^2^	12.20 ^ab^ (5/41)	6.25 ^a^ (2/32)	25.49 ^b^ (13/51)	9.68 ^ab^ (3/31)	0.06
Number of alleles ^3^	2.32	2.15	2.33	2.17	0.46
Mosaicism/mutant ^4^	26.32 (5/19)	15.38 (2/13)	33.33 (13/39)	16.67 (3/18)	0.45
Biallelic KO/total ^5^	5.26 (1/19)	7.69 (1/13)	10.26 (4/39)	11.11 (2/18)	0.91

^a,b^ Values in the same column with different superscripts are significantly different (*p* < 0.05). ^1^ Percentage of embryos with some mutant alleles. ^2^ Percentage of mutant embryos with more than 2 alleles with respect to total embryos. ^3^ Mean number of alleles per embryo. ^4^ Percentage of mutant embryos with more than two alleles with respect to mutant embryos. ^5^ Percentage of mutant embryos with both alleles mutated with respect to total embryos.

**Table 3:** Two-way ANOVA of effect of aphidicolin and methodology (Method).

	Aphidicolin	Method	Aphidicolin × Method
Cleavage rate ^1^	0.39	<0.01	0.83
Blastocyst rate ^2^	0.03	<0.01	0.71
Mutation rate ^3^	0.37	0.53	0.41
Mosaicism/total ^4^	0.03	0.82	0.91
Number of alleles ^5^	0.40	0.63	0.56
Mosaicism/mutant ^6^	0.05	0.85	0.68
Biallelic KO/total ^7^	0.11	0.31	0.69

^1^ Two-cell embryos per total number of inseminated oocytes. ^2^ Blastocysts obtained per total number of inseminated oocytes. ^3^ Percentage of embryos with some mutant alleles. ^4^ Percentage of mutant embryos with more than 2 alleles with respect to total embryos. ^5^ Mean number of alleles per embryo. ^6^ Percentage of mutant embryos with more than 2 alleles with respect to mutant embryos. ^7^ Percentage of mutant embryos with both alleles mutated with respect to total embryos.

**Table 4:** Effect of aphidicolin and methodology on mutation parameters expressed as mean ± SEM.

	E	EAp	M	MAp	*p* Value
Method	Electroporation		Microinjection		
0.5 μM aphidicolin	-	+	-	+	
Mutation rate ^1^	67.44 ± 7.23 (29/43)	52.50 ± 8.00 (21/40)	54.84 ± 9.09 (17/31)	54.17 ± 10.3 (13/24)	0.508
Mosaicism/total ^2^	34.88 ± 7.35 (15/43)	17.50 ± 6.08 (7/40)	32.26 ± 8.53 (10/31)	16.67 ± 7.77 (4/24)	0.177
Number of alleles ^3^	2.47 ± 0.11	2.28 ± 0.10	2.45 ± 0.12	2.42 ± 0.21	0.317
Mosaicism/mutant ^4^	51.72 ± 9.44 (15/29)	33.33 ± 10.54 (7/21)	58.82 ± 12.30 (10/17)	30.77 ± 0.31 (4/13)	0.260
Biallelic KO/total ^5^	11.63 ± 4.94 (5/43)	5.00 ± 3.49 (2/40)	19.35 ± 7.21 (6/31)	8.33 ± 5.76 (2/24)	0.274

^1^ Percentage of embryos with some mutant alleles. ^2^ Percentage of mutant embryos with more than 2 alleles with respect to total embryos. ^3^ Mean number of alleles per embryo. ^4^ Percentage of mutant embryos with more than 2 alleles with respect to mutant embryos. ^5^ Percentage of mutant embryos with both alleles mutated with respect to total embryos.

## Data Availability

Not applicable.
